# *Gnas* Inactivation Alters Subcutaneous Tissues in Progression to Heterotopic Ossification

**DOI:** 10.3389/fgene.2021.633206

**Published:** 2021-01-26

**Authors:** Niambi Brewer, John T. Fong, Deyu Zhang, Girish Ramaswamy, Eileen M. Shore

**Affiliations:** ^1^Department of Orthopedic Surgery, Perelman School of Medicine, University of Pennsylvania, Philadelphia, PA, United States; ^2^Center for Research in FOP and Related Disorders, Perelman School of Medicine, University of Pennsylvania, Philadelphia, PA, United States; ^3^Department of Genetics, Perelman School of Medicine, University of Pennsylvania, Philadelphia, PA, United States

**Keywords:** *Gnas*, heterotopic ossification, progressive osseous heteroplasia, subcutaneous adipose tissue, adipose stromal cells

## Abstract

Heterotopic ossification (HO), the formation of bone outside of the skeleton, occurs in response to severe trauma and in rare genetic diseases such as progressive osseous heteroplasia (POH). In POH, which is caused by inactivation of *GNAS*, a gene that encodes the alpha stimulatory subunit of G proteins (Gsα), HO typically initiates within subcutaneous soft tissues before progressing to deeper connective tissues. To mimic POH, we used conditional *Gnas*-null mice which form HO in subcutaneous tissues upon *Gnas* inactivation. In response to *Gnas* inactivation, we determined that prior to detection of heterotopic bone, dermal adipose tissue changed dramatically, with progressively decreased adipose tissue volume and increased density of extracellular matrix over time. Upon depletion of the adipose tissue, heterotopic bone progressively formed in those locations. To investigate the potential relevance of the tissue microenvironment for HO formation, we implanted *Gnas*-null or control mesenchymal progenitor cells into *Gnas*-null or control host subcutaneous tissues. We found that mutant cells in a *Gnas*-null tissue environment induced a robust HO response while little/no HO was detected in control hosts. Additionally, a *Gnas*-null tissue environment appeared to support the recruitment of control cells to heterotopic bone, although control cell implants were associated with less HO formation compared to mutant cells. Our data support that *Gnas* inactivation alters the tissue microenvironment to influence mutant and wild-type progenitor cells to contribute to HO formation.

## Introduction

The formation of extra-skeletal bone within soft tissues, known as heterotopic ossification (HO), is a frequent patho-physiological response to severe tissue trauma such as combat blast injuries, high impact trauma, and hip replacements (Meyers et al., 2019). However, understanding the cellular mechanisms that precipitate the earliest events of this ectopic bone formation has been challenging. Genetic diseases of HO provide opportunities to identify cellular pathways that direct the altered regulation of cell fates that lead to the formation of cartilage and bone in tissues where they normally do not form, as well as to elucidate properties of these tissues that promote and are permissive for the ectopic development of bone tissue.

Rare genetic disorders caused by heterozygous inactivating mutations of the *GNAS* locus, including progressive osseous heteroplasia (POH), are associated with HO that forms within the skin (Shore et al., 2002; Adegbite et al., 2008; Turan and Bastepe, 2015). In POH, this subcutaneous HO progresses into deeper connective tissues over time (Kaplan et al., 1994; Aynaci and Mu, 2002; Pignolo et al., 2015). Both endochondral and intramembranous mechanisms of ossification have been observed in POH patient lesions (Adegbite et al., 2008; Pignolo et al., 2015; Ware et al., 2019). Activating mutations of the *GNAS* locus are associated with fibrous dysplasia, a disorder of weakened skeletal structure (Zhao et al., 2018), further highlighting the critical role of the *GNAS* locus in the formation and maintenance of bone tissues.

A primary protein product of the *GNAS* (*Gnas* in mice) gene locus is Gsα, the ubiquitously expressed alpha stimulatory subunit of G-proteins. Within mesenchymal progenitor cells, such as adipose stromal cells (ASCs) that reside in adipose tissue, Gsα reciprocally regulates adipogenesis and osteogenesis, with high levels of signal associated with adipogenesis and low levels with osteogenesis (Pignolo et al., 2011; Fan et al., 2012; Liu et al., 2012). Gsα signaling also plays a role in homeostatic mechanisms of the skeleton (Wu et al., 2011; Sinha et al., 2014, 2016; Zhao et al., 2018; Ramaswamy et al., 2018; Ramaswamy et al., 2017), skin and hair follicles (Iglesias-Bartolome et al., 2015).

Mouse models with either germline or conditional cell-specific inactivation of *Gnas* have demonstrated that *Gnas* inactivation induces subcutaneous and progressive HO (Pignolo et al., 2011; Regard et al., 2013). We have adapted such *Gnas* knockout mice to reliably develop subcutaneous post-natal HO over a defined time period in conditional *Gnas*-null mice in order to investigate the early stages of HO initiation and formation. Of note, although POH in patients and *Gnas* inactivation-induced HO in mouse models are associated with heterozygous inactivating mutations of *GNAS*/*Gnas*, the sporadic and mosaic occurrence of sites of HO in patients has led to the suggestion that the regions of HO are caused by “second hits” through DNA damage that result in homozygous inactivation and an additional decrease in *Gnas* expression and/or signaling (Cairns et al., 2013). While this hypothesis has not been confirmed in human disease, conditional or local homozygous knockout of *Gnas* in animal models have shown that ablation of *Gnas* induces increased HO at the targeted sites relative to heterozygous *Gnas* deletion (Cairns et al., 2013; Regard et al., 2013).

In this study, we identified *Gnas* inactivation-induced alterations of the dermal and inguinal subcutaneous adipose tissue where heterotopic bone subsequently forms. We have further demonstrated the influence of the tissue environment on both *Gnas*-null and control progenitor cells to promote aberrant osteogenesis in these tissues.

## Materials and Methods

### Animals

All animal experiments were performed in accordance with the regulations and guidelines and were approved by the Institutional Animal Care and Use Committee (IACUC), University of Pennsylvania.

*R26-CreERT2* mice (Jackson Laboratories, stock no. 008463; Ventura et al., 2007) were crossed to *Gnas*^*fl/fl*^ (Chen et al., 2005; Sakamoto et al., 2005) mice to generate *R26-CreERT2;Gnas^*fl/*+^* mice which were subsequently crossed with *Gnas*^*fl/fl*^ mice to obtain *R26-CreERT2;Gnas^*fl/fl*^* mice. *Ai9*^*fl/fl*^ mice (Jackson Laboratories, stock no. 007909; Madisen et al., 2010) were crossed with *R26-CreERT2;Gnas^*fl/fl*^* mice to generate *R26-CreERT2; Gnas^*fl/*+^; and Ai9^*fl/*+^* mice which were subsequently crossed with *R26-CreERT2;Gnas^*fl/fl*^* mice. Cre-negative mice from the same litters were used as controls. All mice develop normally prior to Cre activation. Global Cre-recombination was induced in 4-week-old mice via intraperitoneal injection of tamoxifen (Sigma) dissolved in corn oil (1 mg/100 μl) on 3 consecutive days (Madisen et al., 2010). Local recombination was induced in 4-week-old mice via subcutaneous injection of 4-hydroxytamoxifen (Sigma) in corn oil (0.3 mg/mL) for 3 consecutive days. Both males and females were used in this study.

### *In vivo* Microcomputed Tomography

Longitudinal *in vivo* microcomputed tomography (μCT) imaging was performed at indicated timepoints using a VivaCT40 (Scanco, Nokomis, FL, United States) at a source voltage of 55 kV, a source current of 145 μA, and an isotropic voxel size of 38.0 μm. Mice were imaged under isoflurane anesthesia. Only the lower limb was imaged to reduce radiation exposure from multiple scans. Three-dimensional images were reconstructed using Scanco microCT V6.1 software. For quantification, the heterotopic bone was reconstructed separately from skeletal bone, with a lower threshold of 220 Hounsfield and an upper threshold of 1,000 Hounsfield units (Culbert et al., 2014).

### Histology

At time of tissue harvest, mice were euthanized and shaved. Hindlimbs from cell implant studies were dissected and fixed in 4% paraformaldehyde for 48 h, decalcified in 10% EDTA for 7 days, washed in 10% sucrose, and embedded in OCT for cryosectioning. Dorsal skin and inguinal white adipose tissues (iWATs) were harvested, fixed in 4% paraformaldehyde, and then paraffin embedded. Sections of 7 μm thickness were cut and stained with hematoxylin and eosin (H&E), Alcian Blue and Orange G, Goldner’s trichrome, or picrosirius red by standard procedures, then imaged under brightfield light microscopy (Nikon Eclipse 90i Upright Microscope). Picrosirius red staining was visualized under polarized light (Leica DMLP Polarizing Microscope).

### Quantification

Image J software was used for all measurements taken. Dermal white adipose tissue (dWAT) thickness area (tissue between epidermal hair follicles and panniculus carnosus muscle) was quantified and averaged over at least 2 mm per sample. Adipocyte cross sectional area was determined using the Fiji Adiposoft plug-in (Galarraga et al., 2012). At least 4 fields of view were analyzed for each sample; *n* = 3–5 samples per genotype per timepoint. Percent area of collagen content was averaged from 20 fields of view at 10x magnification per sample with *n* = 3–5 samples per genotype per timepoint.

### Immunohistochemistry

Cryo-sections from cell implant studies were detected for tissue non-specific alkaline phosphatase (ALP; 1:300, ab65834, Abcam) and the nuclei were co-stained with DAPI. Imaging was performed using a Nikon Eclipse 90i Upright Fluorescence Microscope.

### Cell Implants

Donor adipose-derived stromal cells (ASCs) were isolated from 4-week-old *R26-CreERT2; Gnas^*fl/fl*^;* and *Ai9^*fl/*+^* and R26-*CreERT2; Gnas^+/+^;* and *Ai9^*fl/*+^* control mice (Pignolo et al., 2011). Briefly, inguinal white adipose was dissected, minced using the two-scalpel method, treated with collagenase (Sigma) for 1 h at 37°C, filtered through 100 μm mesh, recovered by centrifugation, and plated in growth medium (DMEM/F12 with 20% FBS and 2% Antibiotic-Antimycotic). Media was changed after 24 h, and adherent cells were expanded and maintained in DMEM/F12 with 10% FBS and 1% Antibiotic-Antimycotic. Recombination was induced *in vitro* with 1 μM 4-hydroxytamoxifen (4-HT, Sigma) 48 h prior to implantation; control cells were also treated with 4-HT. ASCs were genotyped and checked for Ai9 expression via fluorescent imaging prior to implant. 4-week-old host animals (*R26-CreERT2;Gnas^*fl/fl*^*) were treated with 3 consecutive daily subcutaneous injections of 4-HT, as described above, to both hindlimbs; 6 weeks after 4-HT treatment, 400,000 cells were suspended in 100 μL of Matrigel and implanted subcutaneously into hindlimbs (Culbert et al., 2014). Implanted mice were imaged via *in vivo* μCT at the indicated timepoints as described above.

### Statistical Analysis

Statistical analysis was performed using GraphPad (La Jolla, CA, United States) Prism 7 and two-way analysis of variance (ANOVA) with Bonferroni *post-hoc* tests. Values of *p* < 0.05 were considered significant.

## Results

### Postnatal Homozygous Deletion of *Gnas* Induces Heterotopic Ossification *in vivo*

**FIGURE 1:**
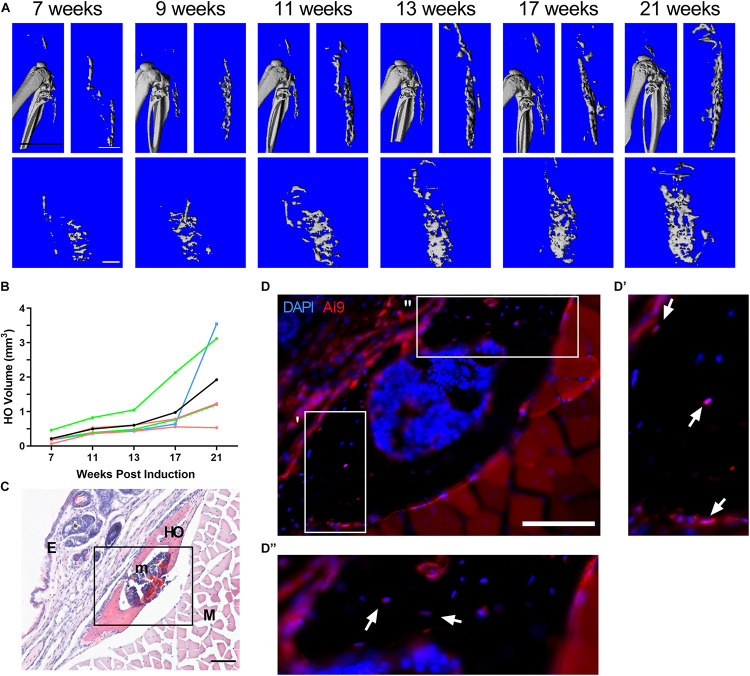
Homozygous deletion of Gnas induces subcutaneous heterotopic ossification *in vivo*. *R26-CreERT2;Gnas^*fl/fl*^;Ai9^*fl/*+^* mice (at 4 weeks old) treated with local hindlimb injection of tamoxifen were examined for ectopic bone formation post-*Gnas* deletion. **(A)** Images of a representative series of longitudinal 3D *in vivo* μCT scans at 7 through 21 weeks post-inactivation. Top – Sagittal view with and without skeletal bone. Bottom – Coronal view of ectopic bone. White scale bar = 1 mm, black scale bar = 5 mm. **(B)** Quantified volumes of HO (*n* = 5); the volume of HO in each animal (raw data provided in Supplementary Table 1) was normalized to the HO volume detected at 7 weeks. Colors – individual mice, Black – average. **(C)** Representative histological section of subcutaneous HO at 21 weeks post-induction (H&E); scale bar = 100 μm. HO, heterotopic ossification (mineralized bone); E, epidermis; m, bone marrow; and M, muscle. **(D)** Immunofluorescent imaging of HO region (box in panel **C**) to detect Ai9^+^;*Gnas*-null cells; nuclei were co-stained with DAPI. Boxes in **(D)** indicate areas with multiple Ai9-negative cells within bone, shown at higher magnification in **(D′** and **D′′)**. Ai9 positive (arrows) and negative cells also appear along the bone surface. Scale bar = 100 μm.

Heterozygous deletion of *Gnas* in a mouse model forms HO slowly, over several months, with unpredictable timing and locations (Pignolo et al., 2011). To facilitate *in vivo* investigations of HO initiation, we developed an approach using a mouse model with inducible postnatal homozygous deletion of *Gnas* that reliably forms heterotopic bone over a shorter time period. At 4 weeks old, *R26-CreERT2; Gnas^*fl/fl*^; Ai9^*fl/*+^* (referred to here as *Gnas*^*fl/fl*^) and control (Cre-negative littermates) mice were treated with subcutaneous injection of 4-hydroxytamoxifen (4-HT) to the hindlimb to activate localized Cre expression and induce *Gnas* deletion (referred to as *Gnas-*null). The floxed tdTomato reporter (*Ai9^*fl/*+^*) allowed tracking of recombined cells within the tissue. Animals were followed longitudinally over a 21-week post-*Gnas* inactivation time course through *in vivo* microcomputed tomography (μCT) imaging at the indicated timepoints (Figure 1). Mineralized ectopic bone was detected by 7 weeks post-inactivation in all *Gnas-*null animals. In all *Gnas-*null animals, the volume of HO increased over the experimental timeline (Figures 1A,B). HO was not detected in vehicle-injected limbs of *Gnas*^*fl/fl*^ mice nor in control mice treated with 4-HT, indicating that depletion of *Gnas* is sufficient to induce HO in this model. Histological analysis at the terminal time point (21 weeks post-inactivation) revealed nodules of HO formation throughout the subcutaneous tissue (Figure 1C) that included Ai9^+^;*Gnas*-null cells (Figure 1D). This localization is consistent with HO initiation sites in POH patients (Kaplan et al., 1994; Aynaci and Mu, 2002; Pignolo et al., 2015). While many cells lining the surface of the ectopic bone were Ai9 positive, both Ai9-positive and Ai9-negative cells were within the ectopic bone (Figures 1D–D′′), indicating the presence of unrecombined and recombined *Gnas-*null cells within the HO and suggesting that cells without *Gnas* inactivation can contribute to the heterotopic bone.

### Deletion of *Gnas* Alters White Adipose Tissues

**FIGURE 2:**
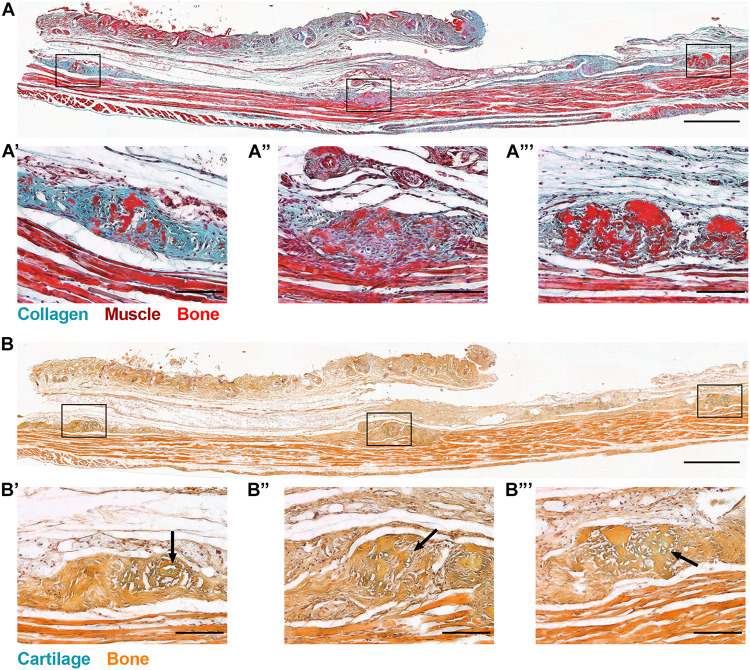
Global *Gnas* inactivation induces subcutaneous heterotopic ossification. *R26-CreERT2;Gnas^*fl/fl*^;Ai9^*fl/*+^* mice (at 4 weeks old) injected intraperitoneally with tamoxifen were examined for ectopic bone formation. At 8 weeks post-induction, skin tissue sections from *Gnas*-null mice were stained with **(A)** Goldner’s trichrome (blue-green, Collagen; brown, Nuclei; red, Muscle; and bright red, Bone), and **(B)** Alcian Blue (cartilage) and Orange G (bone); scale bar = 500 μm. Higher magnification images of HO deposits throughout the skin section highlight excess collagen **(A′–A′′′)** and matrix proteins commonly associated with chondrogenesis within HO regions (**B′–B′′′**, arrows); scale bar = 100 μm; *n* = 3.

In order to define the tissue context of HO initiation, we conducted a histological examination of the subcutaneous tissues that contained HO deposits at the time of μCT detection (Figure 1). Global inactivation of *Gnas* was achieved through intraperitoneal tamoxifen injections of 4-week-old control and *Gnas*^*fl/fl*^ animals. At 8 weeks post-*Gnas* inactivation, islands of HO were detected within the skin of *Gnas-*null animals (Figure 2). In regions where mature dWAT would normally be present, Goldner’s trichrome staining (Figures 2A–A′′′) detected increased collagen (Figure 2A, blue-green staining) in *Gnas-*null skin between the epidermal layer and the panniculus carnosus muscle, as well as in association with HO deposits (Figures 2A′–A′′′, bright red staining). Alcian Blue and Orange G staining of this tissue for cartilage and bone, respectively, (Figure 2B), identified areas of glycosaminoglycan deposits (Figures 2B′–B′′′, arrows, blue staining) associated with HO, suggesting that the HO formation could involve a cartilage intermediate.

**FIGURE 3:**
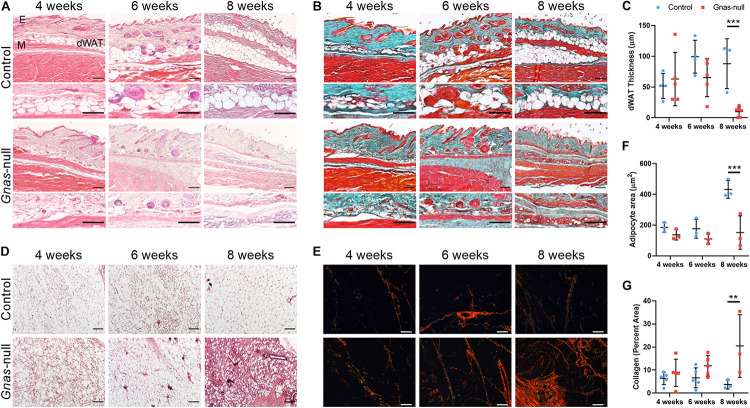
Progressive changes in adipose tissues are induced by global *Gnas* depletion. **(A,B)** Serial sections of skin from control and *Gnas*-null mice at 4, 6, and 8 weeks post-tamoxifen treatment were stained with hematoxylin and eosin **(A)** or Goldner’s Trichrome (green, Collagen; brown, Nuclear chromatin; and red, Muscle, **B**). E, epidermis; dWAT, dermal white adipose tissue; and M, panniculus carnosus muscle. **(C)** Quantification of dWAT thickness. **(D,E)** Inguinal white adipose tissue sections from control and *Gnas*-null mice at 4, 6, and 8 weeks post-tamoxifen treatment were stained with hematoxylin and eosin **(D)** or picrosirius red (collagens) under polarized light **(E)**. **(F)** Quantification of adipocyte cross sectional area. **(G)** Quantification of percent area of collagen fibers under polarized light. Scale bar in all panels, 100 μm; representative images from *n* = 5 (4 and 6 weeks) and *n* = 3 (8 weeks) animals per genotype per timepoint are shown. 2-way ANOVA with Bonferroni *post-hoc* tests **p < 0.01, ***p < 0.001, and relative to control.

In addition to ectopic bone formation, a prominent loss of dWAT within the skin was evident. To interrogate the timeline of *Gnas* inactivation effects on the skin tissue organization prior to HO formation, animals were assayed at 4-, 6-, and 8-weeks post-inactivation. Skin from control and *Gnas-*null animals was stained with hematoxylin and eosin (Figure 3A) or Goldner’s trichrome (Figure 3B) to examine morphological and structural changes. Control animals show the expected tissue structure of a distinct dWAT layer with mature lipid-laden adipocytes. In *Gnas-*null tissue, differences relative to controls were noted beginning at 4 weeks post-inactivation. *Gnas-*null dWAT tissue thickness, in contrast to control, is neither increased nor sustained over the timepoints assayed; rather we detected a statistically significant progressive decrease to near complete depletion by 8 weeks post-inactivation (Figure 3C). The decreased dWAT was accompanied by a progressive increase in detected collagens (Figure 3B, blue-green). Hedgehog (Hh) signaling, a key pathway in skeletal (Day and Yang, 2008) and hair follicle (Mill et al., 2003) development, has previously been implicated in *Gnas* inactivation-induced ectopic ossification (Regard et al., 2013). Consistent with this, we detected ectopic expression of Gli2 between the epidermal layer and the panniculus carnosus muscle at 8-weeks post-*Gnas* inactivation (Supplementary Figure 1), a location and time when we consistently detected HO formation by histology and μCT imaging (Figure 2). Gli2 was also detected at 4- and 6-weeks post-inactivation within the dWAT layer prior to other evidence of ectopic osteogenesis (Supplementary Figure 1).

In addition to dWAT, other white adipose tissue depots were altered by *Gnas* inactivation. Over time, iWAT showed progressively increasing infiltrating fibrotic tissue (Figure 3D) and a significant decrease in mature adipocyte size by 8 weeks post-inactivation (Figure 3F). Picrosirius red staining visualized under polarized light (Figure 3E) revealed increased collagen density within this adipose tissue (Figure 3G). We did not detect HO formation within iWAT at the time points examined but cannot exclude that HO develops within these deep adipose tissue depots over a longer time period.

### A *Gnas*-Null Tissue Environment Promotes Ectopic Bone Formation

Adipose stromal cells are mesenchymal stem cells (MSCs) that reside in adipose tissue (Schäffler and Büchler, 2007), and *in vitro* studies have previously demonstrated that decreased *Gnas* signaling increases osteogenic potential and decreases adipogenic potential of ASCs (Pignolo et al., 2011; Liu et al., 2012). To investigate whether *Gnas-*null progenitor cells are sufficient for the formation of heterotopic bone, we subcutaneously implanted *Gnas-*null or control (*Gnas^+/+^; R26-CreERT2; and Ai9^*fl/*+^)* ASCs into control host tissues. The ASCs additionally carried the floxed Ai9 reporter to fluorescently track the implanted cells; host mice were Ai9 negative. Control and *Gnas*^*fl/fl*^ ASCs were treated with 4-hydroxytamoxifen *in vitro* prior to implantation into the hindlimbs of control mice. Control ASCs implanted into control hosts did not form HO, as expected. Additionally, no HO was detected by *in vivo* μCT imaging when *Gnas-*null ASCs were implanted into control hosts, even at 15 weeks post-implant (Supplementary Figure 2A). Histological analysis verified that Ai9^+^ ASCs remained present within the dWAT (Supplementary Figure 2B). This finding indicates that *Gnas-*null ASCs are insufficient, or inefficient, in cell autonomously differentiating to form HO in a wild-type tissue environment at the timepoints assessed and suggests that a *Gnas*-depleted tissue microenvironment may be relevant to HO formation.

**FIGURE 4:**
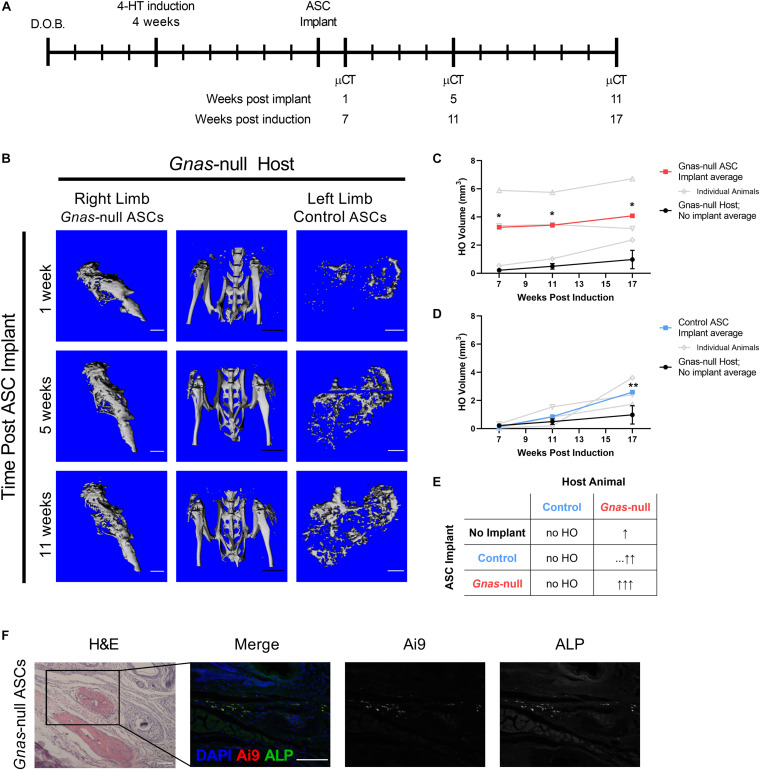
Control and *Gnas*-null ASC contribution to HO formation is dependent on tissue environment. Control and *Gnas*-null adipose stromal cells (ASCs) were implanted into hindlimbs of *Gnas*-null host mice to assess contributions to HO volume. **(A)** Schematic of experiment timeline: host *Gnas* inactivation was induced at 4 weeks of age, ASCs were implanted subcutaneously at 6 weeks post-induction, limbs were imaged by longitudinal *in vivo* μCT at 1, 5, and 11 weeks post-implant (*n* = 3). **(B)** *In vivo* μCT images of hindlimbs from a representative *Gnas*-null host (center), and HO from *Gnas*-null (right limb), and control (left limb) ASC implants over time. White scale bar = 1 mm; black scale bar = 5 mm. **(C)** Quantified HO volumes from *Gnas*-null ASC implants. **(D)** Quantified HO volumes from control ASC implants. In **(C,D)**, HO volumes from individuals (open symbols, gray) and the average of all animals (filled squares, bold) are shown relative to average of HO volumes formed in *Gnas*-null hosts with no cell implants (black filled circles, *n* = 5). 2-way ANOVA with Bonferroni *post-hoc* tests *p < 0.05, **p < 0.01 relative to the baseline HO volume average of *Gnas*-null hosts with no ASC implants at 17 weeks. Raw HO volumes displayed in Supplementary Table 1. **(E)** Summary of HO formation following Control or *Gnas*-null implants compared to *Gnas*-null animals with no implant (top row, No Implant). The number of up arrows indicate relative HO volumes, with dashes (…) indicating a delayed increase in HO. **(F)** Representative images of endpoint histology of Ai9^+^ *Gnas-*null ASC implants into *Gnas*-null hosts stained with H&E, and co-detected for Ai9 and Alkaline Phosphatase (ALP). Scale bar = 100 μm.

To investigate the influence of the *Gnas-*null tissue microenvironment on ASCs, *Gnas-*null and control Ai9^+^ ASCs were isolated and treated as described above, and implanted subcutaneously into the right and left hindlimbs, respectively, of host Ai9-negative *Gnas-*null mice at 6 weeks post-inactivation of *Gnas* (Figure 4A). This implant timepoint is just prior to detection of ectopic mineralized tissue in host *Gnas*-null animals (Figure 1A). To detect HO, *in vivo* μCT was performed longitudinally at 1-, 5-, and 11-weeks post-implantation (7-, 11-, and 17-weeks post-*Gnas*-inactivation of hosts; Figure 4B). HO volumes in *Gnas-*null hosts that did not receive implants were quantified as baseline values for comparison (Figures 4C,D; black lines).

*Gnas-*null ASCs implanted into *Gnas-*null hosts formed significantly more HO, as early as 1-week post implant (7 weeks post host animal inactivation), relative to *Gnas-*null animals that did not receive implants (Figure 4C and Supplementary Table 1). Control ASCs implanted into *Gnas-*null hosts also showed significantly more volume relative to *Gnas-*null animals that did not receive implants (Figure 4D), although the onset of increased heterotopic bone formation appeared delayed relative to the *Gnas-*null ASC implant response. These data are summarized in Figure 4E. Histological examination at the 11-week post-implant endpoint showed that Ai9^+^ cells were present, within and adjacent to HO deposits in *Gnas-*null host tissues (Figure 4F). Detection for ALP, a marker of differentiating osteoblasts (Vimalraj, 2020), indicated co-localization of ALP with Ai9^+^ cells (Figure 4F) supporting their participation in HO formation. Taken together, these results strongly support that *Gnas* inactivation in cells of the host tissues where HO initiates alters these tissues to provide a tissue environment that is conducive to supporting MSC osteogenesis.

## Discussion

Previous studies have shown that decreased *Gnas* signaling increases osteogenic potential of progenitor cells that reside within adipose tissue (Pignolo et al., 2011). In this study, our data support that *Gnas* inactivation induces HO not only by conferring an enhanced osteogenic differentiation potential to mesenchymal progenitor cells that likely participate in forming heterotopic bone, but also by altering the properties and maintenance of the tissues within which the ectopic bone develops.

We determined that *Gnas* inactivation induces significant changes to the tissues where HO will eventually begin to form. A system of postnatally-induced inactivation of *Gnas* allowed us to monitor progressive tissue changes over time, pre-, and post-HO formation, in response to the loss of *Gnas*. Over time, and prior to detection of HO by histology or μCT imaging, the dermal adipose tissue layer thickness and adipocyte size progressively decreased. In place of lipid-laden adipocytes, this region of the tissue gained a more fibrotic appearance and showed increasingly dense staining for collagens, tissue properties that are likely to contribute to a stiffer, more osteogenic-supportive environment (Levental et al., 2009; Cox and Erler, 2011).

A shift from a soft adipose tissue microenvironment to a stiffer ECM fiber-rich environment void of lipid-laden adipocytes is expected to alter the biomechanical signaling within the tissue (Humphrey et al., 2014; Shoham et al., 2014). The YAP/TAZ-mediated mechanosignaling pathway as well as activation of RhoA mechanosignaling are pro-osteogenic signals that influence gene transcription and cytoskeletal shape, respectively, (McBeath et al., 2004; Dupont et al., 2011). Signaling by Gsα, has been linked to regulation of the mechanotransductive factors YAP and TAZ, with Gsα activation of cyclic-AMP resulting in cytoplasmic localization and degradation of YAP and TAZ (Yu et al., 2012), and suggesting that *Gnas*/Gsα inactivation would promote YAP/TAZ signaling and a pro-osteogenic state.

Recent studies in mouse models of fibrodysplasia ossificans progressiva (FOP), another rare genetic disorder of HO, have established a precedent for key roles of the tissue microenvironment and biomechanical signaling in supporting and promoting HO (Haupt et al., 2019; Stanley et al., 2019). The FOP *Acvr1*^R206H^ mutation was shown to alter the physical properties of the tissue where HO will form, producing more collagens and exhibiting increased tissue stiffness. Further, *Acvr1*^R206H^ progenitor cells show aberrant upregulation of both RhoA and YAP/TAZ biomechanical signaling pathways, which are associated with osteogenic differentiation (Haupt et al., 2019; Stanley et al., 2019).

Although the cellular mechanisms through which the tissue microenvironment and mechano-signaling could promote *Gnas*-mediated HO require further investigation, recent studies have shown positive interactions between mechanotransductive pathways and Hh/Gli2 pathway activation during epidermal homeostasis (Akladios et al., 2017), and PKA, a downstream effector of active Gsα signaling, acts as a negative regulator of Hh signaling (Riobó et al., 2006). Increased Hh pathway activation has previously been established as an important regulator of *Gnas* inactivation-induced HO (Regard et al., 2013; Xu et al., 2018). In our *Gnas*-null model of HO, we detected progressively increased Gli2^+^ cells in the subcutaneous tissues prior to evidence of mineralized tissue formation and surrounding regions of mineralized HO. This suggests that increased Hh signaling may influence the initiation of osteogenesis, perhaps in part by altering mechanosignaling, and may serve as a marker for developing HO lesions.

In our model of HO, we present evidence that the tissue microenvironment in *Gnas*-null mice is important in supporting HO formation. Although *Gnas* mutant ASCs have increased osteogenic capacity *in vitro* (Pignolo et al., 2011), when implanted into a normal/control *in vivo* subcutaneous microenvironment, *Gnas*-null ASCs appeared insufficient to induce HO. In contrast, both control and *Gnas*-null ASCs that were introduced into a *Gnas*-null tissue environment, at a time prior to HO detection as determined by μCT, were recruited to participate in subsequently formed ectopic bone.

We note that not all cells within the detected heterotopic bone are Ai9^+^ implanted cells; likely, endogenous Ai9^–^ progenitor cells in the *Gnas*-null hosts are recruited to bone formation prior to the implantation timepoint, since *Gnas-*null hosts form HO in the absence of any cell implants. Regardless, the implanted *Gnas*-null ASCs were associated with more rapid and robust heterotopic bone formation than the control cell implants when compared to the baseline volume of HO formation in control *Gnas*-null animals without implanted ASCs. However, the apparent delayed increase in HO formation in response to implanted control ASCs versus *Gnas*-null ASCs suggests that mutant progenitor cells are more readily recruited to HO formation. These data suggest that ASC contribution to HO development may not require their reduced *Gnas* expression, with wild-type cells recruited to ectopic bone formation through non-cell autonomous signals from the *Gnas*-null tissue microenvironment. Hh signaling has been shown to be at least one pathway that is disrupted by Gsα-mediated signaling during the formation of HO (Regard et al., 2013). Given their critical roles in bone growth and maintenance, additional signaling pathways that are likely to be relevant to HO formation include the YAP/TAZ (discussed above) and BMP (relevant to FOP HO as noted above) pathways.

The POH mouse model used in our current study formed subcutaneous HO through either local subcutaneous inactivation of *Gnas* or through global/systemic inactivation, suggesting that the HO may be more dependent on local effects of *Gnas* inactivation. However, we note that POH is classified among the pseudohypoparathyroidism (PHP) disorders, caused by deficiencies in *Gnas*/Gsα and/or downstream signaling effectors, some of which are accompanied by resistance to PTH and/or other hormones and by altered serum biochemistries (Linglart et al., 2018; Mantovani and Elli, 2018). Although endocrine abnormalities have not been associated with POH (Adegbite et al., 2008; Linglart et al., 2018), the POH mouse model provides an opportunity to more closely investigate whether changes in endocrine function accompany the progressive formation of HO and this will be examined in future studies.

A limitation of our current study is that the *in vivo* population of HO progenitor cells has not yet been clearly defined. Here, we used primary ASCs isolated from adipose tissue as a proxy, and potential candidate, for endogenous HO progenitors based on previous work demonstrating the influence of *Gnas* inactivation on their differentiation potential (Pignolo et al., 2011; Liu et al., 2012) and because HO deposits in our model are found highly associated with adipose tissue. Mesenchymal cell populations are present in a variety of tissues proximal to HO deposits including skin (Fujiwara et al., 2018), adipose (Merrick et al., 2019), and muscle (Contreras et al., 2019), and remain other potential candidates that may contribute to the formation of subcutaneous HO.

Our study focused on dWAT since this is the site where HO typically initiates in POH patients (Kaplan et al., 1994; Aynaci and Mu, 2002; Pignolo et al., 2015). However, our examination of iWAT, a larger hypodermal depot relative to dWAT, determined that *Gnas*-null iWAT was similarly diminished with corresponding increased detection of collagens, indicating a general role of *Gnas* in maintaining adipose tissues. Notably, we did not detect HO formation in iWAT during the time course examined. This may be attributed to the relative size differences of iWAT and dWAT depots, resulting in a longer time to transition the adipose tissue to be sufficient to support HO formation in iWAT. Alternatively, differences in dWAT and iWAT function, structure, and/or rates of tissue turnover could also determine the presence of HO or rate of formation (Chen et al., 2019).

In our histological examination of early stage HO, regions of developing HO were positive for glycosaminoglycans, suggesting associated chondrogenesis. HO lesions in POH appear to be most commonly formed through intramembranous ossification, while endochondral ossification is apparently less frequent although has been noted in patient biopsies (Adegbite et al., 2008; Ware et al., 2019). The *in vitro* chondrogenic potential of *Gnas*-null progenitor cells has not been investigated and more discrete timepoints in our mouse model are needed to identify the timing and mechanism of chondrogenesis.

Our data support that HO is a complex process that requires coordinated interactions among multiple cells and tissues. Progenitor cells that can be directed toward chondro/osteogenic fates are clearly required, and data support a cell-autonomous mechanism of *Gnas* inactivation in these progenitor cells. However, our results additionally provide evidence that cell non-autonomous signals that alter the tissue microenvironment where HO forms are required to establish a conducive environment for the differentiation and development to ectopic bone tissue. Whether the progenitor cells themselves are the source of both cell autonomous and non-autonomous signals, or other cells within neighboring tissues contribute key inductive factors, or both, remains to be established. Ongoing investigations using tissue and cell specific Cre-drivers to inactivate *Gnas* will help to determine how specific cell populations contribute to subcutaneous and progressive HO.

While non-hereditary, injury-induced HO is a frequent secondary complication of hip replacement surgery, head and spinal cord injuries, deep tissue burns, and other forms of severe trauma (Pignolo and Foley, 2005), the cellular mechanisms and changes to tissues that are permissive for ectopic bone formation to initiate have been difficult to identify. Genetic diseases of HO have now identified specific genes and pathways that direct HO and have permitted the development of genetic animal models to investigate the processes that promote the aberrant formation of ectopic bone tissue. The use of FOP models has revealed critical stages prior to HO formation for intervention such as increased tissue stiffness (Haupt et al., 2019), immune cell infiltration (Convente et al., 2018), and chondrogenesis (Chakkalakal et al., 2016). Similarly, our study presents a new system to model subcutaneous HO initiation and formation, and an opportunity to provide novel insights into understanding initiating factors for HO development and additional possibilities for treatment strategies for both genetic and non-genetic forms of HO formation.

## Data Availability Statement

The original contributions presented in the study are included in the article/Supplementary Material, further inquiries can be directed to the corresponding author/s.

## Ethics Statement

The animal study was reviewed and approved by Institutional Animal Care and Use Committee (IACUC) University of Pennsylvania.

## Author Contributions

NB, JF, GR, and ES designed the study and analyzed the data. NB, JF, and DZ performed the experiments. NB and ES wrote the manuscript. All authors contributed to the article and approved the submitted version.

## Conflict of Interest

The authors declare that the research was conducted in the absence of any commercial or financial relationships that could be construed as a potential conflict of interest.
