# Supplementary material for: Gnas Inactivation Alters Subcutaneous Tissues in Progression to Heterotopic Ossification
Source: Front Genet. 2021 Jan 26;12:633206. doi: 10.3389/fgene.2021.633206 (PMC7870717; doi:10.3389/fgene.2021.633206)
Supplement: Supplementary file 1 [file Data_Sheet_1.docx]

Supplementary Material

*Gnas* inactivation alters subcutaneous tissues in progression to heterotopic ossification

Niambi Brewer^1,2,3^, John T. Fong^1,2^, Deyu Zhang^1,2^, Girish Ramaswamy^1,2^, Eileen M. Shore^1,2,3*^

^1^ Department of Orthopaedic Surgery, Perelman School of Medicine, University of Pennsylvania, Philadelphia, PA, USA

^2^ Center for Research in FOP and Related Disorders, Perelman School of Medicine, University of Pennsylvania, Philadelphia, PA, USA

^3^ Department of Genetics, Perelman School of Medicine, University of Pennsylvania, Philadelphia, PA, USA

*** Correspondence:**Corresponding Author
shore@pennmedicine.upenn.edu

Keywords: Gnas, progressive osseous heteroplasia (POH), heterotopic ossification (HO), subcutaneous adipose tissue, adipose stromal cells

**
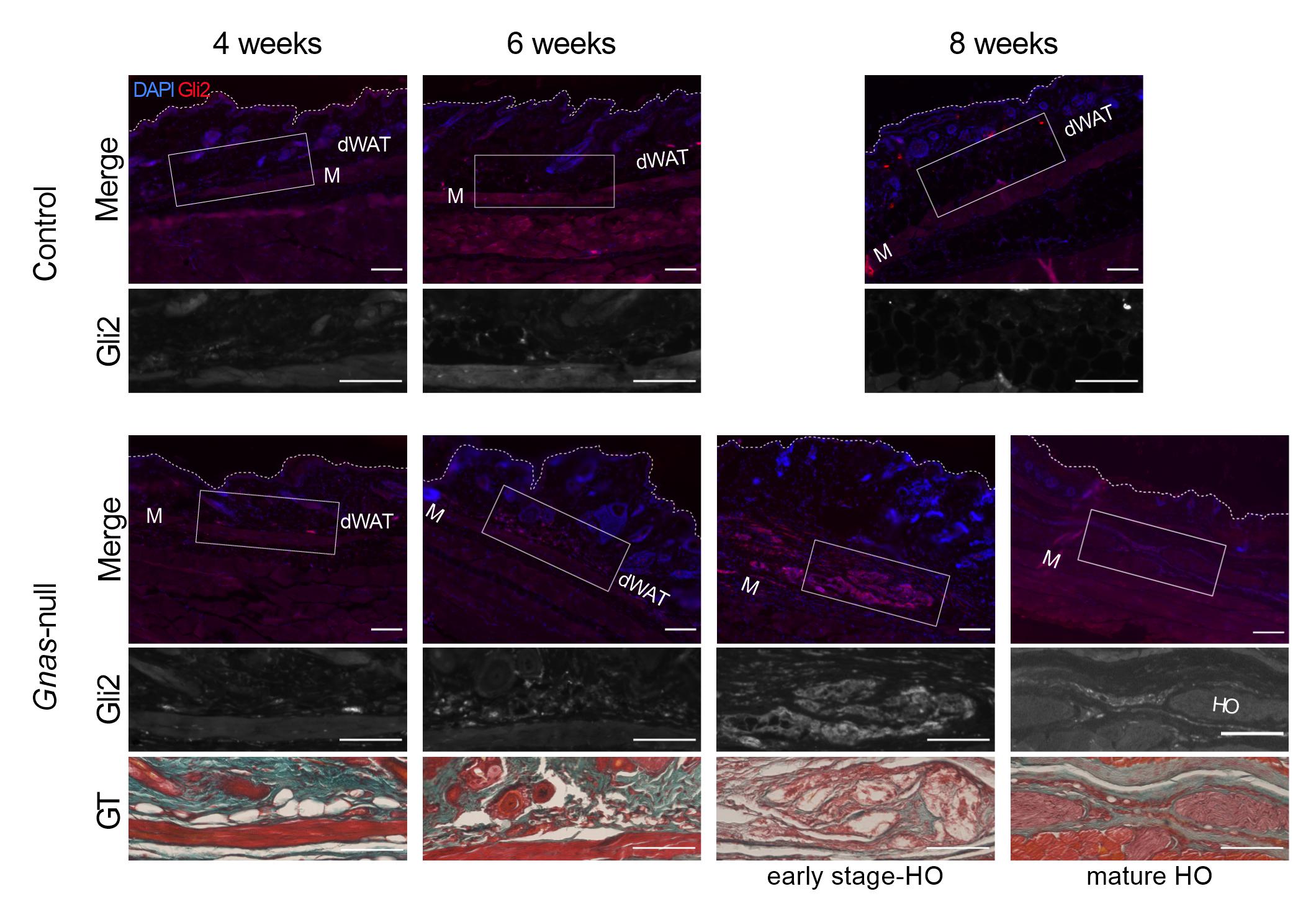
**

**Supplementary Figure 1.** *Hedgehog signaling is induced in dWAT regions prior to detection of HO*.

Sections of dorsal skin from control and *Gnas*-null mice at 4, 6, and 8 weeks post-tamoxifen treatment were detected by immunohistochemistry for the Hedgehog pathway marker Gli2 (1:250, AF3635, R&D Systems). Nuclei were co-stained with DAPI. Dotted line, epidermal layer; dWAT, dermal white adipose tissue; M, panniculus carnosus muscle; HO, heterotopic ossification. Boxes indicate regions shown at higher magnification in Gli2 IHC and Goldners-Trichrome (GT) stained panels. (GT staining: collagens, blue-green; bone, red; muscle, bright red) Scale bar = 100 µm; representative images from n=5 (4 and 6 weeks) and n = 3 (8 weeks) animals per genotype per timepoint are shown. For the assessment of Gli2 detection in the absence of discernible dermal white adipose tissue (dWAT) in 8-week *Gnas*-null samples, we examined regions between hair follicles and panniculus carnosus muscle. Note that in *Gnas*-null tissues, Gli2 was associated with regions of adipose depletion, collagens, and nascent HO (early-stage HO), with increased Gli2 detection over time. The GT-stained panel at far right shows a more mature mineralized region of HO from 8-weeks; Gli2 was detected mainly in the area between the mineralized HO, likely associated with less mature osteoprogenitors.

**
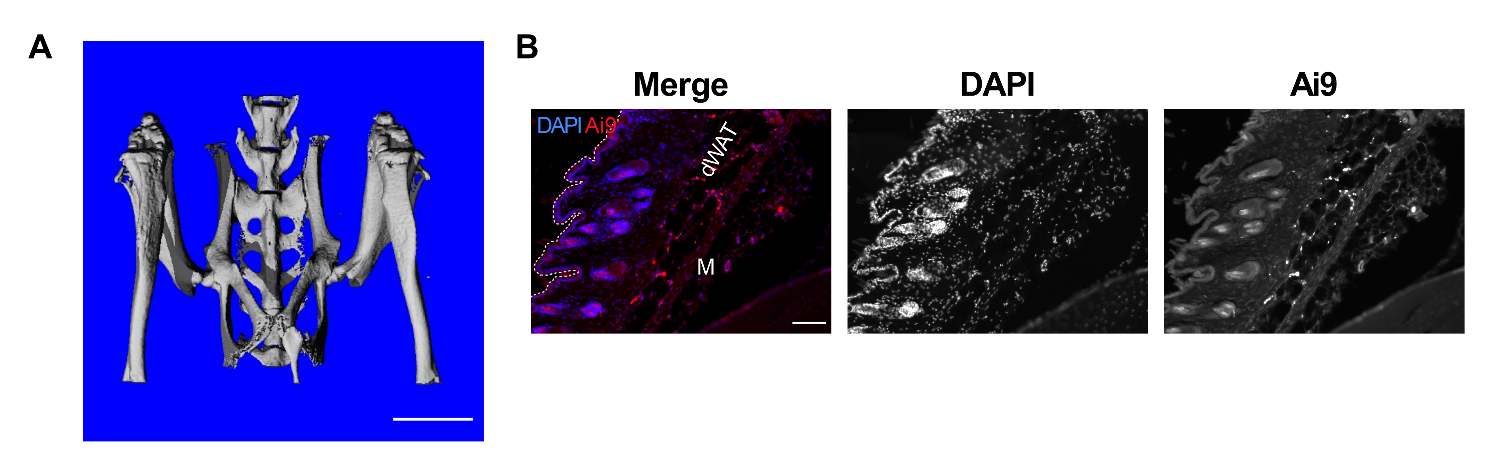
**

**Supplementary Figure 2.** *Gnas-null ASCs are insufficient to form ectopic bone in a control host tissue environment*.

*Gnas*-null Ai9^+^ ASCs were implanted into the hindlimbs of control animals and assayed for HO formation. (A) Representative *in vivo* µCT image of a control host at 15 weeks post-implant with *Gnas*-null ASCs. No ectopic bone was detected. Scale bar = 5 mm; n=4. (B) Histological analysis by fluorescent microscopy of tissues from the cell implant location that were stained with DAPI (nuclei) showed that Ai9^+^ implanted cells were resident within the subcutaneous tissue at 15 weeks post implant. Scale bar = 100 µm. Dotted line, epidermal layer; dWAT, dermal white adipose tissue; M, panniculus carnosus muscle.

|  | ***Gnas-*null Host, no implant**  (n=5) | | | | | ***Gnas-*null**  **ASC implant** | | | **Control**  **ASC implant** | | |
| --- | --- | --- | --- | --- | --- | --- | --- | --- | --- | --- | --- |
|  |  |  |  |  |  | **676-R** | **680-R** | **683-R** | **676-L** | **680-L** | **683-L** |
| **7 weeks** | 0.1704 | 0.0653 | 0.1996 | 0.4615 | 0.1785 | 5.8908 | 3.374 | 0.5391 | 0.0454 | 0.3287 | 0.0427 |
| **11 weeks** | 0.5293 | 0.3604 | 0.3893 | 0.826 | 0.3675 | 5.748 | 3.4664 | 1.0292 | 0.8151 | 1.5408 | 0.1989 |
| **17 weeks** | 0.7854 | 0.5548 | 0.7667 | 2.1333 | 0.6396 | 6.7201 | 3.1751 | 2.3633 | 1.7321 | 2.4109 | 3.6192 |

**Supplementary Table 1.**  *Raw HO volumes for Figure 4 data.*

HO volumes were quantified over time by *in vivo* uCT. All values are mm^3^. HO volumes were quantified in *Gnas*-null host mice (n=5, one outlier removed via Grubbs test) at 7, 11, 17 weeks post-induction of *Gnas* inactivation to provide baseline HO formation data in the absence of cell implants. *Gnas*-null host mice (n=3) were implanted with *Gnas*-null adipose stromal cells (ASCs) into the right (R) hindlimb and control ASCs into the left (L). 676, 680, and 683 are mouse ID numbers.
